# Comparison of Photofermentative Hydrogen Production in Cylindrical Photobioreactors Using Different Mixing Systems

**DOI:** 10.3390/microorganisms13061386

**Published:** 2025-06-14

**Authors:** Raffaella Margherita Zampieri, Eleftherios Touloupakis, Cecilia Faraloni, Isabela Calegari Moia

**Affiliations:** 1 Department of Agriculture, Food, Environment and Forestry, University of Florence, Via San Bonaventura 13, 50145 Firenze, Italy; raffaellamargherita.zampieri@unifi.it; 2 Research Institute on Terrestrial Ecosystems, National Research Council, Via Madonna del Piano 10, 50019 Sesto Fiorentino, Italy; isabelacalegarimoia@cnr.it; 3 Institute of BioEconomy, National Research Council, Via Madonna del Piano 10, 50019 Sesto Fiorentino, Italy; cecilia.faraloni@ibe.cnr.it

**Keywords:** photosynthetic bacteria, photofermentation, cell culture, renewable energy, light conversion efficiency

## Abstract

In this work, the ability of the photosynthetic purple non-sulfur bacterium *Rhodopseudomonas* sp. to produce H_2_ was investigated in two cylindrical photobioreactors (PBRs). The PBRs used in this work had different working volumes: 0.2 L of working volume (named 0.2-PBR) and 4.0 L of working volume (named 4.0-PBR). Two mixing methods were tested in the 4.0-PBR. The first used a rotor with four paddles, and the second a spiral rotor. Additionally, light conversion efficiency (LCE) was assessed for the three conditions tested. The culture in the 0.2-PBR produced 142.15 mL of H_2_ with an average H_2_ production rate of 0.74 mL/h, an average productivity of 3.70 mL/L/h and an LCE = 0.59%. The culture in the 4.0-PBR produced a total of 806.05 mL and 1642 mL of H_2_ with the paddle rotor and the spiral rotor, respectively. The average H_2_ production rate and LCE of the two rotors were 2.29 mL/h and LCE = 0.58% in the case of the paddle rotor and 2.87 mL/h and LCE = 0.72% in the case of the spiral rotor. The more uniform and thus more efficient mixing of the cells achieved with the spiral rotor played an important role compared to the paddle rotor, resulting in a higher LCE. This study presents a scale-up from 0.2 L to 4.0 L of the photofermentation process using the purple non-sulfur bacterium *Rhodopseudomonas* sp. S16-VOGS3.

## 1. Introduction

Increasing economic growth, population growth and technological progress have led to higher energy consumption worldwide. Fossil fuel reserves are limited, so the research community is under increasing pressure to find new sustainable energy sources. In response, the idea of a greener circular economy based primarily on renewable resources has gained traction [1]. Microbes for bioprocessing and waste streams as potential substrates/feedstocks are an important strategy to address energy and environmental issues and reduce dependence on fossil fuels [2]. With the innate advantage of producing green energy through the cultivation of photosynthetic microorganisms, green energy is expected to play an important role in the global economy and help mitigate environmental problems such as global change caused by fossil fuel consumption [3].

These microorganisms, which thrive in both terrestrial and aquatic environments, play an important role in the production of bioproducts by sequestering CO_2_ through photosynthesis. Through intensive cultivation, photosynthetic microorganisms can be used to produce commercial green energy such as biodiesel and molecular hydrogen (H_2_) [3,4]. H_2_ is a very efficient, environmentally friendly and renewable fuel that can be produced by dark fermentation and photofermentation by a variety of facultative and obligate anaerobic bacteria as well as purple bacteria and microalgae [5,6]. Molecular H_2_ is very promising due to its high conversion efficiency, recyclability and environmentally friendly properties [7]. The production of H_2_ by bacteria using low-cost and commercially available substrates such as crude glycerol or various industrial, agricultural and other carbonaceous materials represents a very promising sustainable technological approach [8]. Biohydrogen production from biomass or organic waste also helps with waste management and benefits environmental sustainability and energy production. Photosynthetic microorganisms, including microalgae, cyanobacteria and purple bacteria, are particularly promising candidates for light-dependent H_2_ production [3,9]. Biological H_2_ is produced through processes such as dark fermentation, microbial fermentation and algae-based techniques, all of which have the potential to drastically reduce greenhouse gas emissions.

Purple Non-Sulfur Bacteria (PNSB) are Gram-negative photosynthetic bacteria that grow under anaerobic or microaerobic conditions, typically observed as red colonies. They are often used as model organisms to study the metabolic control of nitrogen and carbon metabolism [10]. Thanks to their high metabolic adaptability, they are found in a variety of environments and can survive under different conditions. PNSB are anoxygenic photoautotrophs capable of light-dependent CO_2_ fixation and phototrophic energy transfer [11]. Depending on light, carbon and oxygen levels, they can grow as photoautotrophs (presence of light and CO_2_), photoheterotrophs (presence of light and organic compounds), or chemoheterotrophs (presence of organic compounds) [12]. They have no oxygen-generating activity, absorb solar energy in the visible and near infrared wavelength range and have a high substrate conversion efficiency. Photophosphorylation and oxidative phosphorylation are the two major energy metabolisms associated with both the transmembrane transfer of protons and electron transport [12]. PNSB can utilize various organic carbon compounds including pyruvate, acetate and other organic acids, amino acids, alcohols and carbohydrates [13]. PNSB can also utilize aromatic organic compounds as a carbon source [14].

**Figure 1:**
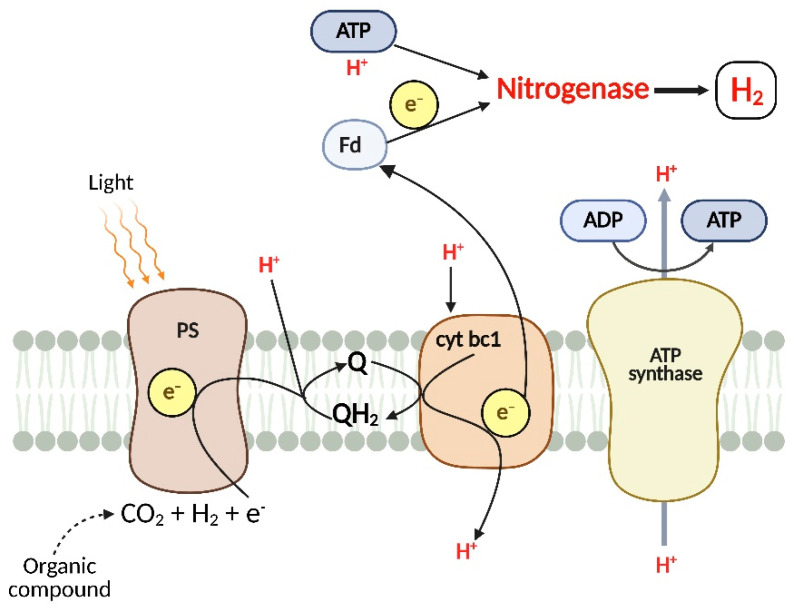
Schematic representation of H_2_ production via photofermentation by photosynthetic bacteria. PS: photosystem; Q: Quinone; QH_2_: ubihydroquinone; cyt bc1: cytochrome c oxidoreductase; Fd: ferredoxin.

PNSB convert anaerobically organic substrates into molecular H_2_ in a process known as photofermentation [15] (Figure 1). For example, *Rhodobacter sphaeroides*, *Rhodopseudomonas palustris* and *Rhodospirillum rubrum* can utilize organic acids for H_2_ production with the nitrogenase enzyme [14,16,17]. Photofermentative H_2_ production requires oxygen-free and ammonia-limited conditions, as nitrogenase is inhibited by the presence of oxygen and ammonium salts [15]. Under these conditions, nitrogenase catalyzes H_2_ formation at the cost of four moles of adenosine triphosphate (ATP) according to the reaction: 8H^+^ + 8e^−^ + 16ATP → 4H_2_ + 16ADP + 16P_i_ [15]. The tricarboxylic acid cycle uses the carbon supply to generate electrons and carbon dioxide, which initiates the process of photofermentation. Subsequently, electron carriers such as nicotinamide adenine dinucleotide (NAD/NADH) and ferredoxin (Fd)_ox_/(Fd)_red_ are successively oxidized and reduced to deliver the electrons to the nitrogenase. To generate H_2_, the ATP produced by the photosynthetic process is transferred to the nitrogenase together with protons and electrons [15]. The most used nitrogen source for photofermentative H_2_ is glutamate, as it has low nitrogenase inhibition and is rapidly consumed [3,18,19].

A variety of photosynthetic bacteria such as *R. sphaeroides* and *R. palustris*, along with organic acids such as acetate and lactate, have been used for photofermentation [20,21]. Numerous articles have discussed the production of photofermentative H_2_ in the field and in the laboratory [9,22,23]. The photofermentative H_2_ production rate depends on the cell growth conditions such as the composition of the growth medium, the light, temperature and pH conditions [15,24]. The photofermentation process can be enhanced by the addition of chemicals to the growth medium, such as nitrogenase co-factor (e.g., iron, molybdenum and nickel), ethylenediaminetetraacetic acid (which increases the solubility and availability of metals) and yeast extract [25].

Photosynthetic bacteria have been investigated for the synthesis of H_2_ using wastewater, single organic acids or mixtures of organic acids as carbon sources [21,26,27,28]. The type of carbon source affects the efficiency of H_2_ production, which is due to the different electron transfer capabilities of photosynthetic bacteria that differ in their metabolic pathway. Waste materials such as molasses, cheese whey and olive mill effluent have been used to feed photosynthetic bacteria [8,29,30]. Co-cultures with purple photosynthetic bacteria and dark fermentative bacteria have also been investigated to produce H_2_ [31,32].

To be commercially successful, sustainable H_2_ must be produced with photosynthetic bacteria grown in efficient photobioreactors (PBRs). The PBRs provide an optimized cultivation environment for microbial growth and H_2_ production by regulating factors such as temperature, light intensity and nutrient availability [33,34]. PBRs are typically operated at relatively high cell densities, resulting in significant light attenuation due to absorption and scattering by the cells. Therefore, proper agitation of the culture is essential to ensure a homogeneous distribution of nutrients, temperature and light throughout the suspension, boosting the productivity [33].

Research is needed to optimize H_2_ production with a focus on PBR design, cost-effective technologies and the scale-up process.

In this work, we investigated the photofermentative H_2_ production by *Rhodopseudomonas* cells in two different PBRs. The PBRs were cylindrical and had two different working volumes of 0.2 L (0.2-PBR) and 4.0 L (4.0-PBR), with the aim of investigating the response of *Rhodopseudomonas* H_2_ productivity to the scale-up process. In addition, two different types of rotors were used to stir the culture in the 4.0-PBR, exploring the effect of the mixing on the cultures.

## 2. Materials and Methods

### 2.1. Growth Conditions

The bacterium *Rhodopseudomonas* sp. S16-VOGS3 (hereafter *Rhodopseudomonas*), from the culture collection of the National Research Council’s Research Institute on Terrestrial Ecosystems, Florence, Italy, was used in this study. The 16S sequence of *Rhodopseudomonas* sp. S16-VOGS3 was deposited in GenBank under the following accession numbers: KU899101 KU899105. The *Rhodopseudomonas* cells were pre-cultured using a modified van Niel growth medium. The medium contained 6 g/L acetate, 0.5 g/L NH_4_Cl, 1 g/L KH_2_PO_4_, 0.4 g/L NaCl, 0.4 g/L MgSO_4_ 7H_2_O, 0.05 g/L CaCl_2_ 2H_2_O, 0.1 mg/L p-aminobenzoic acid, 0.005 g/L ferric citrate and 10 mL/L mineral solution for micronutrients. The mineral solution (1 L) contained 1 mg CuCl_2_ 2H_2_O, 2 mg NiCl_2_ 6H_2_O, 3 mg MnCl_2_ 4H_2_O, 10 mg ZnSO_4_ 7H_2_O, 20 mg CoCl_2_ 6H_2_O, 30 mg H_3_BO_3_, 200 mg FeSO_4_ 7H_2_O and 500 mg Na_2_MoO_4_ 7H_2_O. The growth medium and the bioreactors were sterilized by autoclaving for 20 min at 121 °C and 1.0 atm pressure in a FALC ATV1100 autoclave (FALC Instruments srl, Treviglio, Italy). Cultures were continuously illuminated using a power-star HQI-TS OSRAM halogen lamp (OSRAM, Munich, Germany) (80 W/m^2^) and maintained at 30 °C. In order to remove the growth medium, the *Rhodopseudomonas* culture was centrifuged (3000 rpm for 10 min) in a Sorvall Super T21 centrifuge (Thermo Fisher Scientific Inc., Waltham, MA, USA) and the pellet was washed twice with sterile saline solution (NaCl 0.9%). The cells were then resuspended in the H_2_ production medium (Section 2.3).

### 2.2. Photobioreactors Set-Up

#### 2.2.1. The 0.2-PBR

**Figure 2:**
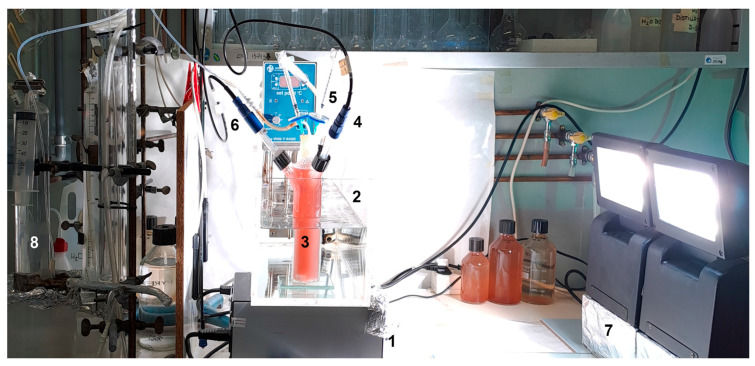
The 0.2-PBR setup used for the cultivation of *Rhodopseudomonas*. (1) magnetic stirrer; (2) water bath; (3) photobioreactor; (4) pH sensor; (5) inlet for sterile HCl solution; (6) ORP probe; (7) lamp; (8) calibrated glass column.

The 0.2-PBR is a cylindrical Pyrex glass bottle (15.9 cm height, 4 cm inner diameter, 200 mL working volume) with a flat bottom. It has a main opening at the top, which is sealed with a silicone stopper fitted with a Tygon tube for the outflow of H_2_ and two side openings for the pH and oxidation-reduction potential (ORP) electrodes (Figure 2). A needle for the addition of sterile HCl solution is inserted into the silicone stopper. The PBR is placed in a M900-TI thermostatic water bath (MPM Instruments srl, Bernareggio, Italy) at 30 °C and the cells are mixed using a magnetic bar and a Falc F30 magnetic stirrer (Falc Instruments srl, Treviglio, Italy). Illumination with a total intensity of 75 W/m^2^ is provided on one side of the PBR by a Power star HQI-TS OSRAM halogen lamp (OSRAM, Munich, Germany).

#### 2.2.2. The 4.0-PBR

**Figure 3:**
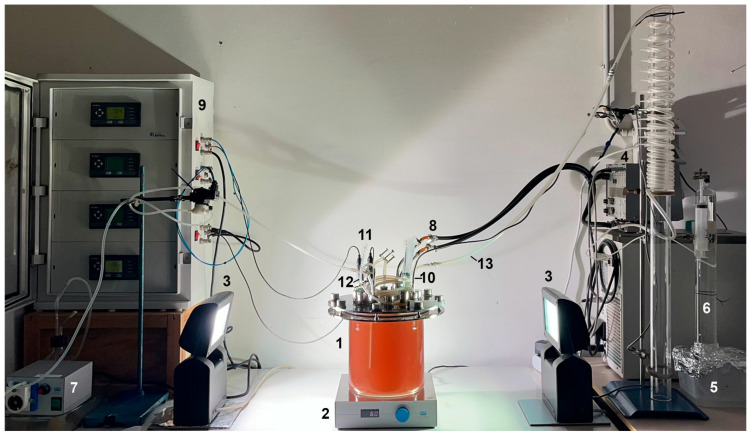
The 4.0-PBR setup used for the cultivation of *Rhodopseudomonas* (1) equipped with an internal 4-paddle cylindrical shaped device; (2) magnetic stirrer; (3) lamps; (4) cryostat; (5) saline solution basin; (6) calibrated column trap; (7) pump for pH control; (8) heating/cooling finger; (9) control unit; (10) temperature probe; (11) pH and ORP probes; (12) sample port; (13) gas outlet.

**Figure 4:**
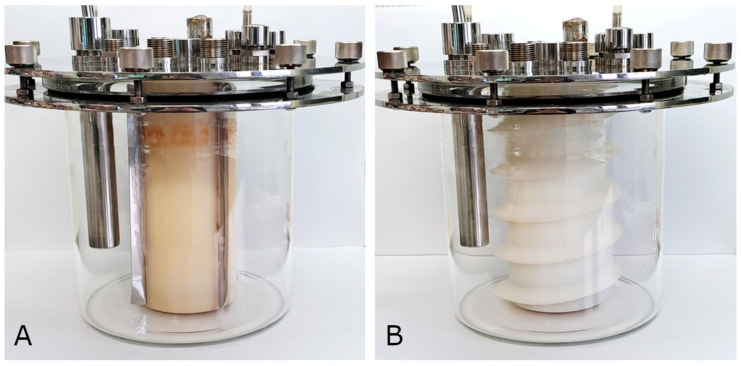
The two different rotor types used in this study in the empty 4.0-PBR. (**A**) The rotor with 4-paddles and (**B**) the spiral rotor.

The 4.0-PBR is a cylindrical 4-litre glass system (Figure 3). The reactor consists of a cylindrical culture chamber (17.5 cm high and 19.4 cm inner diameter, 4.0 L working volume) with an internal rotor (20 cm high and 9.1 cm outer diameter). The bottom of the rotor is equipped with a magnet and a MSL 25 magnetic stirrer (VELP Scientifica Srl, Usmate, Italy) underneath ensures its rotation (60 rpm) and thus the mixing of the culture. Two different rotor types were used in this study, a rotor with 4-paddles and a spiral rotor (Figure 4). The culture chamber has a lid with a dowel to center the rotor and a heat exchanger to control the culture temperature (30 °C); this heat exchanger is connected to an external refrigerated-heating circulator (Julabo, Seelbach, Germany). It is also equipped with a series of holes in which the pH, ORP and temperature probes are positioned, a tube for taking culture samples, two tubes for the inflow and outflow of gases, and a tube for the inflow of acidic solution for pH control. A control unit (Chemitec srl, Florence, Italy) measures both the redox potential and the pH of the culture; the latter parameter was maintained in the range of 7.2 by adding sterile HCl solution (10 mM) using an SP311 peristaltic pump (VELP Scientifica Srl, Usmate, Italy). The outer surface of the reactor is illuminated by two opposite lamps (OSRAM Power star 150 Watt HQI-TS lamps), each providing an intensity of 75 W/m^2^.

### 2.3. H_2_ Production

H_2_ production by *Rhodopseudomonas* cells was performed under anaerobic conditions with both PBRs filled with a growth medium containing 6.0 g/L acetate and 1.0 g/L glutamate instead of NH_4_Cl (C/N ≈ 30). A calibrated column immersed in a CO_2_ absorber solution was used to collect the gas produced by the culture. Before each experiment, the cells in culture were incubated in the dark for 24 h to achieve anaerobiosis.

### 2.4. Analytical Procedures

A Quantum Radiometer-Photometer model LI-250A (LICOR, Lincoln, NE, USA) was used to measure the irradiance on the outer surface of the PBR. Biomass cell dry weight (CDW) was determined by measurements performed in triplicate according to the following protocol [28]. Samples of 5 mL were taken from the culture filtered through pre-weighed Whatman GF/F filters with 0.7 mm pore size (Merck, Darmstadt, Germany). The filters with the cells were washed twice with deionized water. They were then dried in an MPM Instruments type M60-VN oven (MPM Instruments srl, Bernareggio, Italy) at 70 °C for 16 h and weighed in a PBI model bc analytical balance (VWR International (PBI) Srl, Milan, Italy). Acetate was determined spectrophotometrically using the Spectroquant^®^ Volatile Organic Acids Test (Merck, Darmstadt, Germany).

The gas produced by the culture was analyzed using a Clarus 500 gas chromatograph (PerkinElmer, Waltham, MA, USA) with a Carbosieve SII Spherical Carbon packed column (Supelco. Inc., Bellefonte, PA, USA) and a thermal conductivity detector. Gas chromatography was conducted under the following operating conditions: an isothermal program at 35 °C for 2.25 min, with N_2_ as the carrier gas set to a flow rate of 30 mL/min; the injection and detector temperatures were set to 150 °C.

Poly(3-hydroxybutyrate) (PHB) was determined in the form of crotonic acid by high performance liquid chromatography (HPLC). We used 5.0 mL of culture for acid digestion to crotonic acid by boiling it at 106 °C in 1.0 mL of pure sulfuric acid for 30 min. This treatment converts PHB into crotonic acid, which was assayed by HPLC. The latter was performed by using an HPLC-Thermo Finnigan System 6000LP equipped with a Synergi-Hydro-RP C-18 column (250 × 4.6 mm i.d.) (Phenomenex, Torrance, CA, USA) and a UV detector (214 nm). A mobile phase comprising 15% (*v*/*v*) acetonitrile and 0.1% (*v*/*v*) H_3_PO_4_ in aqueous solution was employed using a flow rate of 0.8 mL/min.

The light conversion efficiency (LCE) was calculated as the following ratio (energy output)/(energy input) × 100 [28]. The energy output is equal to the energy of the H_2_ produced. The energy input consists of the light irradiance on the surface of the PBRs and the energy of the organic molecules consumed. We considered (i) 12.94 J/mL as the energy content of H_2_ at 25 °C; (ii) the light irradiance on the PBR surface, calculated as light intensity (J/m^2^/s) reactor illuminated surface (m^2^), 0.89 (glass transparency, 0.80 in the case of the 4.0-PBR); (iii) 708.8 kJ/mol the heat of combustion of acetate. The irradiated area of the 0.2-PBR was calculated as ½ of the cylindrical reactor surface (2πr_i_h), where r_i_ and h indicate the inner radius and height of the cylindrical reactor, respectively [19]. Due to the geometry of the cylindrical PBR shape, a dilution factor of 1.57 was applied to determine the average effective irradiance incident on the semi-circumference of the PBR. This value represents light dilution due to the curved surface [35]. It is the ratio between the illuminated surface area of the cylindrical reactor (πr_i_ × h) and the basal area (2r_i_ × h) of the reactor, (πr_i_ × h)/(2r_i_ × h) = π/2 = 1.57. The detailed list of calculations is reported in the Supplementary Material file.

## 3. Results

### 3.1. H_2_ Production in the 0.2-PBR

**Figure 5:**
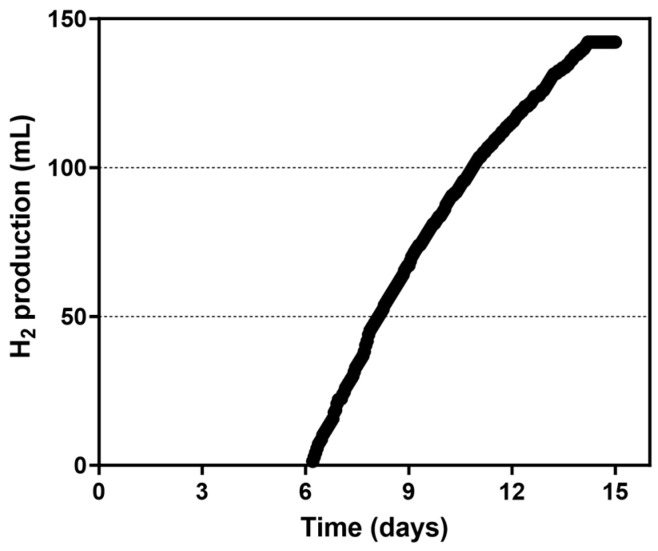
Cumulative H_2_ production by *Rhodopseudomonas* cells grown in 0.2-PBR.

**Figure 6:**
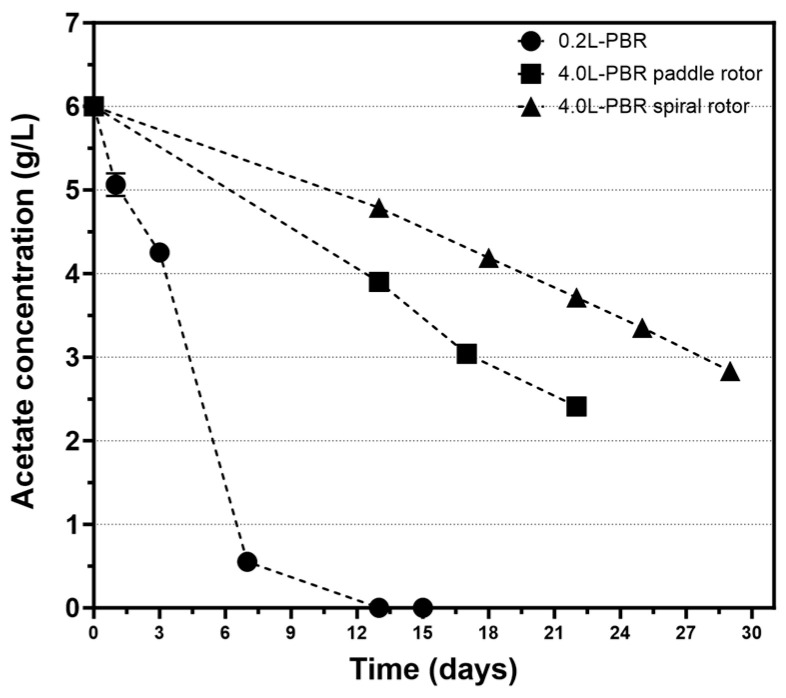
Changes in acetate concentration over time during H_2_ production in the two PBRs.

The starting biomass of 1.926 g/L, equivalent to 0.385 g of CDW, was added to the 0.2-PBR, which was then filled with the medium for H₂ production, sealed and left in the dark for 24 h to support anaerobiosis. The PBR was then placed in the light and the ORP began to decrease until it reached a stable negative value of −530 mV. At this point, photofermentative H_2_ production began which was followed for about 8 days (Figure 5). The cumulative amount of H_2_ produced during the experiment was 142.15 mL, which corresponds to 710.75 mL/L culture. The maximum H_2_ production rate (HPR) was 2.12 mL/h, with an average HPR of 0.74 mL/h and a productivity of 3.70 mL (H_2_)/L (culture)/h or 1.99 μL(H_2_)/mg (cells)/h. During the experiment CDW in the culture increased from 1.926 ± 0.011 to 3.960 ± 0.070 g/L after 15 days of growth, which was in line with acetate consumption. Considering the complete consumption of acetate, an LCE of 0.59% was calculated (Figure 6). Both the irradiance at the surface of the PBR and the consumption of the organic compounds were considered when calculating the total energy input. PHB production by the culture in 0.2-PBR increased from 16.8 ± 0.4 mg/L to 86.6 ± 1.3 mg/L, corresponding to 0.88% and 2.19% of PHB over the CDW, respectively.

### 3.2. H_2_ Production in the 4.0-PBR

#### 3.2.1. Use of a 4-Paddle Rotor

**Figure 7:**
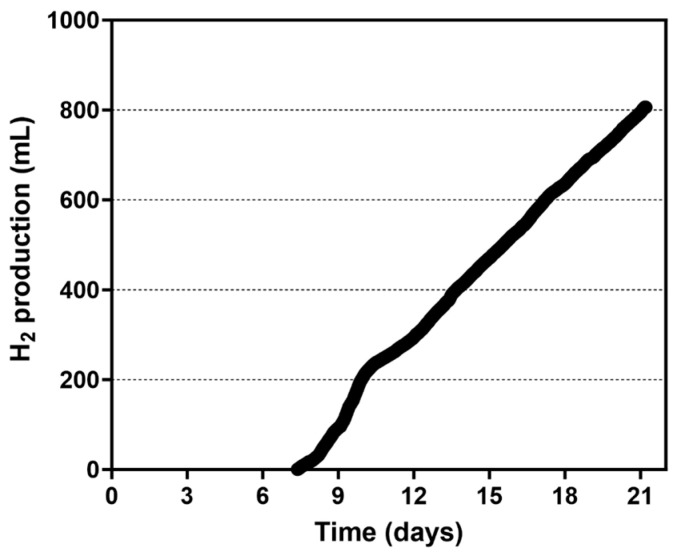
Cumulative H_2_ production by *Rhodopseudomonas* cells grown in the 4.0-PBR using the 4-paddle rotor.

As for the 0.2-PBR, the concentration of *Rhodopseudomonas* at the beginning of the experiment corresponded to 1.92 g/L, equivalent to 7.68 g of CDW. After the addition of the biomass and the medium for H_2_ production, the 4.0-PBR was sealed and left in the dark for 24 h to promote anaerobiosis. The system was then placed in the light and the ORP began to decrease, reaching a stable negative value. At this point, photofermentative H_2_ production began which was followed for about 13.8 days (Figure 7).

The total amount of H_2_ released during the experiment was 806.05 mL, which corresponds to 201.5 mL/L culture. The maximum HPR was 6.21 mL/h, with an average HPR of 2.29 mL/h and a productivity of 0.574 mL (H_2_)/L (culture)/h or 0.299 μL (H_2_)/mg (cells)/h. During the experiment CDW in the culture increased from 1.92 ± 0.08 to 2.90 ± 0.02 g/L after 21 days of growth. Considering the consumption of acetate, from 6.0 g/L to 2.41 ± 0.02 g/L an LCE of about 0.58% was calculated (Figure 6). Both the irradiance at the surface of the PBR and the consumption of the organic compounds were considered when calculating the total energy input. PHB production by the culture in the 4.0-PBR using the paddle rotor increased from 16.8 ± 0.4 mg/L to 62.3 ± 0.4 mg/L, corresponding to 0.88% and 2.15% of PHB over the CDW, respectively.

#### 3.2.2. Use of a Spiral Rotor

**Figure 8:**
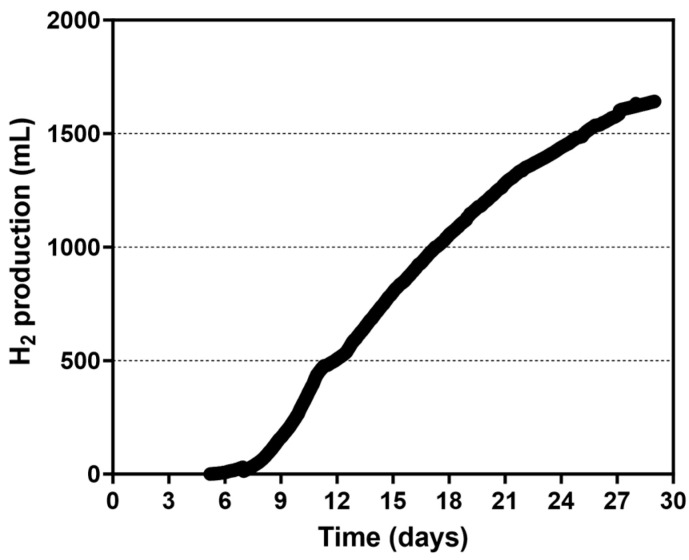
Cumulative H_2_ production by *Rhodopseudomonas* cells grown in the 4.0-PBR using the spiral rotor.

The initial biomass of 7.64 g of CDW, equivalent to 1.91 g/L, was added to the 4.0-PBR, which was then filled with the medium for H₂ production, sealed and left in the dark for 24 h to sustain anaerobiosis. The PBR was then illuminated, leading to the decrease in the ORP until it reached a stable negative value. Subsequently, photofermentative H_2_ production initiated which was followed for about 24 days (Figure 8). The cumulative amount of H_2_ collected during the experiment was 1642 mL, which corresponds to 410.5 mL/L culture. The maximum HPR was 9.03 mL/h, with an average HPR of 2.87 mL/h and a productivity of 0.717 mL (H_2_)/L (culture)/h or 0.375 μL (H_2_)/mg (cells)/h. During the experiment, CDW in the culture increased from 1.91 ± 0.01 to 2.60 ± 0.02 g/L which was in line with acetate utilization that decreased from 6.0 g/L to 2.83 ± 0.09 g/L (Figure 6). Considering the consumption of acetate an LCE of about 0.72% was calculated. Both the irradiance at the surface of the PBR and the consumption of the organic compounds were considered when calculating the total energy input. PHB production by the culture in the 4.0-PBR using the spiral rotor increased from 16.8 ± 0.4 mg/L to 46.3 ± 0.6 mg/L, corresponding to 0.88% and 1.78% of PHB over the CDW, respectively.

## 4. Discussion

The metabolic adaptability of PNSB makes them attractive for a wide range of biotechnological applications. Photofermentation is the process by which PNSB convert anaerobically organic substrates into molecular H_2_ [25].

Photofermentation has been carried out using a range of photosynthetic bacteria including *R. sphaeroides* and *R. palustris*, as well as organic acids like acetate and lactate [20,21]. Numerous articles have been published on the production of photofermentative H_2_ in the field and in the laboratory [9,22,23,34]. The rate of photofermentative H_2_ production is determined by cell growth parameters such as the composition of the growth medium, light, temperature and pH [24]. The addition of chemicals to the growth media, such as nitrogenase cofactors, ethylenediaminetetraacetic acid and yeast extract can improve the photofermentation process [25].

Photosynthetic bacteria have been studied to produce H_2_ using wastewater, single organic acids or combinations of organic acids as carbon sources [21,26,28,34]. The type of carbon source influences the efficiency of H_2_ production, which is due to the different electron-transfer capabilities of photosynthetic bacteria that have different metabolic pathways. The use of waste materials to feed photosynthetic bacteria improves the cost efficiency of the photofermentative process by simultaneously producing H₂ and utilizing waste materials. Several types of wastewater and industrial wastes have already been tested as substrates, including palm oil mill effluent, olive mill waste, molasses, cheese whey, brewery wastewater and winery wastewater [8,29,30,36]. One approach to increase the H_2_ yield is based on the co-cultivation of biological systems to optimize the conversion of organic material. Dark fermentative bacteria transform their substrates into volatile fatty acids, which are then metabolized by PNSB, resulting in the formation of H_2_ [31]. This process can be carried out in two stages, or by integrating mixed cultures, which could be beneficial in terms of both productivity and economics [32]. Several studies have investigated co-cultures of purple photosynthetic bacteria and dark fermentative bacteria to produce H_2_ [37]. Table 1 summarizes recent studies on photofermentative H_2_ production by photosynthetic bacteria.

Scaling up photosynthetic bacteria cultures from a lab scale to a larger scale is challenging because it is difficult to evaluate the factors that influence the expansion process during cultivation. The challenges of scaling up photosynthetic bacteria cultures can usually be reduced to two main issues: high cost and low efficiency. In the case of H_2_ production using solar irradiation, as far as the cost of PBRs is concerned, it is assumed that the materials for construction account for about 35% of the production cost, which increases to 63% if the nutrients for the preparation of the medium are included [38].

Recently, Genç and Koku performed a techno-economic analysis of photofermentative production based on current technology [39]. By adding up the capital and operating costs of the system, they determined an H_2_ cost of 1362 USD/kg H_2_. The most important factors for successful photobiological H_2_ production in large-scale outdoor PBRs are the reactor design in which efficient LCE can be achieved, the use of an efficient mixing system and adequate monitoring and control of culture parameters [40]. In the case of PBRs for H_2_ production, the design becomes more complicated as the reactor must be perfectly sealed to prevent H_2_ losses [3]. Therefore, the cost of H_2_ production could become profitable through the development of genetically modified strains by exploiting advances in synthetic biology and optimizing the usability of sunlight.

**Table 1:** Recent works regarding photofermentative H_2_ production by photosynthetic bacteria.

Organism	PBR Type (Liters)	Carbon Source (g/L)	Light Intensity	Productivity (mL/L/h)	LCE (%)	Reference
*R. sphaeroides HY01*	Composite tubular (70)	Glucose (5.4)	3000 lx	22.8	4.0	[41]
*R. sphaeroides O.U. 001*	Flat plate (0.235)	Malate (2.0)	64 W/m^2^	12.7	-	[42]
*R. capsulatus*	Stirred tank (1.5)	Lactose (10)	5000 lx	3.37	-	[43]
*Rhodospirillum rubrum*	Stirred tank (1.5)	Lactose (10)	5000 lx	6.4	-	[43]
*R. sphaeroides O.U. 001*	Flat panel (1.0)	Malate (2.05)	10.25 W/m^2^	11.0	3.31	[44]
*Rhodopseudomonas* sp.	Cylindrical (0.22)	Acetate (4.0)	80 W/m^2^	10.2	1.20	[28]
*R. capsulatus* DSM 1710	Flat panel (1.4)	Acetate (3.6)	4000 lx	18.0	-	[45]
*R. palustris GCA009*	Flat panel (0.9)	Glucose (10)	210 W/m^2^	30.4	5.34	[46]
*Rhodopseudomonas*	Cylindrical (0.2)	Acetate (4)	80 W/m^2^	14.9	2.37	[19]
*R. sphaeroides O.U.001*	Cylindrical (2.0)	Dark fermentative cheese whey effluent	10,000 lx	41.9	-	[47]
*R. sphaeroides KKU-PS1*	Serum bottles (0.12)	Succinate fermentation effluent	15,000 lx	13.8	3.10	[48]
*Rhodopseudomonas* sp. S16-VOGS3	Cylindrical (0.2)	Acetate (6.0)	75 W/m^2^	10.6	0.59	This work
*Rhodopseudomonas* sp. S16-VOGS3	Cylindrical (4.0) Paddle rotor	Acetate (6.0)	75 W/m^2^	1.55	0.58	This work
*Rhodopseudomonas* sp. S16-VOGS3	Cylindrical (4.0) Spiral rotor	Acetate (6.0)	75 W/m^2^	2.25	0.72	This work

In this work, we investigated the scale-up of the photofermentation process with *Rhodopseudomonas* in two cylindrical PBRs with different working volumes. The most efficient system tested was the 4.0-PBR with the spiral rotor, characterized by an average H_2_ production rate of 2.87 mL/h. The use of the 4-paddle rotor in the same 4.0-PBR resulted in a reduced rate of 2.29 mL/h. Lastly, the rate of the 0.2-PBR was only 0.74 mL/h, clearly indicating the successful outcome of the scale-up process. However, the productivity (10.6 mL (H_2_)/L(culture)/h) obtained in the 0.2-PBR was higher than the results of the 4.0-PBR and the results of previous studies in which different strains were used under different culture conditions (Table 1). The reason can be attributed to the smaller size of the 0.2-PBR, which permits higher availability of light energy for the cells.

The lower H_2_ production yield in the 0.2-PBR can be attributed to the higher PHB production in this culture compared to the 4.0-PBR. It is known that the production of H_2_ and PHB are two metabolic pathways that compete for the assimilation of reducing equivalents [49]. The 0.2-PBR culture had a 1.31% increase in PHB over the CDW, the PHB enhancement in the 4.0-PBR with the 4-paddle rotor corresponded to 1.27%, while only a 0.9% increase in PHB was detected in the 4.0-PBR with the spiral rotor. Despite the extremely low values of these three percentages, it is evident that PHB production is inversely proportional to H_2_ production.

The differences in product yield and LCE between the 4.0-PBR with the spiral rotor and the other two PBRs tested can mainly be attributed to the culture mixture, as other culture parameters such as the geometry of the PBR, growth media, temperature and illumination were the same. The effects of mixing on H_2_ production have hardly been considered so far, making the results of this study an interesting starting point to be addressed in future research. It is known that a light gradient within the culture depth is unavoidable for photosynthetic microorganisms growing in dense cultures, so that the cells are subject to a light/dark cycle that is strongly influenced by mixing. The high concentration of the culture or the design of the PBR can cause the cells to shade with each other and reduce their growth rate. As a result, a good mixing mechanism is required to keep the cells in constant motion and ensure that each cell receives light. The culture must be mixed to obtain a homogeneous cell suspension, optimize light absorption and facilitate gas exchange and nutrient distribution. The turbulence of the liquid repeatedly transports the cells from the darker interior of the reactor to the brighter peripheral zone, to prevent the cells from going without light for long periods.

The intricate link between the growth of photosynthetic bacteria and culture conditions largely explains the difficulty in creating customized PBRs that allow sufficient H_2_ production. Therefore, preliminary studies in a fully specified process in which all relevant parameters can be precisely controlled are crucial. An industrial-scale PBR can be economically viable if the culture parameters have first been defined on a laboratory scale. For instance, efficiency of the culture is determined by the combination of light distribution, transmission and collection. The mutual shading and absorption of light by the cells creates a light gradient within the PBR. Light penetration into a cell culture can be successfully estimated by Beer–Lambert’s law:

$$I_{x}=I_{0}\;e^{-\alpha_{c}C_{b}b}$$

where *α_c_*—the extinction coefficient of the biomass, *C_b_*—the cell biomass concentration, *I*_0_ and *I_x_*—light intensities at the surface and distance *x*, *b*—path length of the light from the surface to any point *x* in the PBR.

The light distribution among the cells is directly dependent on how the culture is mixed. Mixing is critical for cell growth and metabolism as it promotes homogeneity of mass, heat, light distribution and mass transfer. When mixing, it is important to maintain an optimal shear rate that promotes cell growth while minimizing physical stress [50].

Few studies have been performed to improve the efficiency and yield of H_2_ production with photosynthetic bacteria, focusing on culture mixing, as this is an important factor affecting the performance of continuous operation of the H_2_ production systems [44,46,51]. To evaluate the performance of our culture against the existing literature on H_2_ production, we estimated the LCE of the process as the ratio of the energy stored in H_2_ to the incident irradiance on the PBR. Gilbert et al. investigated H_2_ production with *R. sphaeroides* in a flat panel rocking PBR [44]. They solved the problem of agitation in a flat plate reactor by setting the reactor in a rocking motion with a fulcrum in the center. Their studies with *R. sphaeroides* O.U. 001 resulted in a maximum production rate of 11 mL/L/h with a LCE = 3.31%. Wang et al. investigated a flat PBR that included a zigzag grid column to promote mixing. They obtained a maximum H_2_ production rate of 30.4 mL/L/h with an LCE = 5.34% [46]. Hanipa et al. studied H_2_ production with *R. sphaeroides* KKU-PS1 using 0.12 L serum bottles [48]. They achieved a maximum H_2_ production rate of 13.8 mL/L/h with an LCE = 3.1%. Ren et al. investigated H_2_ production with *R. sphaeroides* HY01 using a 70 L tubular PBR. They achieved a maximum H_2_ production rate of 22.8 mL/L/h with an LCE = 4% [41].

The LCE value obtained in the 4.0 L-PBR with the spiral rotor (0.72%) was lower than the values previously reported by our group (1.20%) [28] and Moia et al. (2.37%), who used immobilized *Rhodopseudomonas* cells [19]. Similarly, the LCE obtained was lower than the values reported by Wang et al. (5.34%) [46] using immobilized *R. palustris* GCA009 and by Ren et al. (4.0%) [41] using *R. sphaeroides* HY01, both of which used glucose as the carbon source. The LCE value of our study was also lower than that of Gilbert et al. (3.31%) [44] using *R. sphaeroides* O.U.001 in a flat panel rocking PBR and Hanipa et al. (3.10%) [48] using *R. sphaeroides KKU-PS1* in serum bottles, with malate and succinate as carbon sources, respectively.

In this study, the highest LCE value (0.72%) was achieved using the 4.0-PBR with the spiral rotor and acetate as the carbon source. The use of the spiral rotor in the 4.0-PBR had a positive effect on H_2_ production and LCE compared to the 4-paddle rotor. This is possibly due to the gentler movement generated by the spiral rotor that ensured a more effective mixing of the culture. The shear stress exerted on the cells when using the paddle rotor to mix the cultures had a negative effect on H_2_ production. Our results show that improving culture mixing is one of the technological aspects that can improve the yield of H_2_ production.

## 5. Conclusions

In this study, photofermentative H_2_ production by *Rhodopseudomonas* cells in two types of PBRs was investigated. In addition, the effects of using a new spiral rotor on H_2_ production were evaluated. This work was an attempt to scale up the photofermentation process using two cylindrical PBRs with different working volumes. Of the two mixing systems tested in the 4.0L-PBR, the spiral rotor outperformed the paddle rotor in terms of LCE and H_2_ production rate, achieving 0.72% compared to 0.58% and 2.87 mL/h compared to 2.29 mL/h, respectively. The better performance of the spiral rotor was attributed to its more effective mixing, reduced shear stress and improved light distribution and cell exposure. These results highlight the importance of PBR design and suggest that optimizing the mixing mechanisms can significantly impact the efficiency of H_2_ production in upscaled systems. However, further research is needed to improve their industrial sustainability and commercialization. For example, improving LCE and reducing the costs associated with nutrient supply can make the process more viable.

## Data Availability

The data that support the findings of this study are available from the corresponding author upon reasonable request.

## Supplementary Materials

The following supporting information can be downloaded at: https://www.mdpi.com/article/10.3390/microorganisms13061386/s1. Calculations performed to obtain the light conversion efficiency values.
