# Supplementary material for: Comparison of Photofermentative Hydrogen Production in Cylindrical Photobioreactors Using Different Mixing Systems
Source: Microorganisms. 2025 Jun 14;13(6):1386. doi: 10.3390/microorganisms13061386 (PMC12195309; doi:10.3390/microorganisms13061386)
Supplement: Supplementary file 1 [file microorganisms-13-01386-s001.zip › microorganisms-3661942-supplementary.pdf]

Supplementary material

Comparison of photofermentative hydrogen production in cylindrical photobioreactors using different mixing systems

Raffaella Margherita Zampieri[1,2], Eleftherios Touloupakis[2,*], Cecilia Faraloni[3], Isabela Calegari Moia[2]

[1] Department of Agriculture, Food, Environment and Forestry, University of Florence, Via San Bonaventura 13, 50145, Firenze, Italy;
[2] Research Institute on Terrestrial Ecosystems, National Research Council, Via Madonna del Piano 10, 50019, Sesto Fiorentino, Italy;
[3] Institute of BioEconomy, National Research Council, Via Madonna del Piano 10, 50019, Sesto Fiorentino, Italy;

*Corresponding author: eleftherios.touloupakis@cnr.it

1. Light conversion efficiency calculation

1.1 0.2-PBR

The LCE was calculated as the following ratio (energy output)/(energy input)*100.

The energy output is equal to the energy of the H_2 produced.

The 0.2-PBR produced 142.15 mL of H_2 × 12.94 J/mL (energy content) = 1839.42 J

Energy output = 1839.42 J

The energy input consists of the light irradiance on the surface of the PBRs and the energy of the organic molecules consumed.

The energy of light irradiance on the surface of the 0.2-PBR was calculated as follows:

Light intensity = 75 W/m^2 = 75 J/s/m^2

Time = 192 hours, corresponding to 691200 s

Illuminated surface of the reactor = 99.852 cm^2, corresponding to 0.0099852 m^2

Glass transparency = 0.89

Light dilution = 1.57

Energy of light irradiance = (75 J/s/m^2 × 691200 s × 0.0099852 m^2 × 0.89) / 1.57= 293435.1 J

Energy of the consumed acetate = 1.2 (g)/59 (MW) × 708.8 kJ/mol (heat of combustion of acetate) = 14416.27 J

Energy input = 293435.1 J + 14416.27 J = 307851.3 J

LCE = Energy output / Energy input = 1839.42 / 307851.3 = 0.005975 ≈ 0.59%

1.2 4.0-PBR paddle rotor

The LCE was calculated as the following ratio (energy output)/(energy input)*100.

The energy output is equal to the energy of the H_2 produced.

The 4.0-PBR (paddle rotor) produced 806.05 mL of H_2 × 12.94 J/mL (energy content) = 10430.29 J.

Energy output = 10430.29 J

The energy input consists of the light irradiance on the surface of the PBRs and the energy of the organic molecules consumed.

The energy of light irradiance on the surface of the 4.0-PBR was calculated as follows:

Light intensity = 75 W/m^2 = 75 J/s/m^2

Time = 331 hours, corresponding to 1191600 s

Illuminated surface of the reactor = 355.34 cm^2, corresponding to 0.0355 m^2

Glass transparency = 0.80

Light dilution = 1.57

Energy of light irradiance = (75 J/s/m^2 × 1191600 s × 0.0355 m^2 × 0.80) / 1.57= 1616629.3 J

Energy of the consumed acetate = 14.36 (gr)/59 (MW) × 708.8 kJ/mol (heat of combustion of acetate) = 172514.7 J

Energy input = 1616629.3 J + 172514.7 J = 1789114 J

LCE = Energy output / Energy input = 10430.29 / 1789114 = 0.005829 ≈ 0.58%

1.3 4.0-PBR spiral rotor

The LCE was calculated as the following ratio (energy output)/(energy input)*100.

The energy output is equal to the energy of the H_2 produced.

The 4.0-PBR (paddle rotor) produced 1642 mL of H_2 × 12.94 J/mL (energy content) = 21247.48 J.

Energy output = 21247.48 J

The energy input consists of the light irradiance on the surface of the PBRs and the energy of the organic molecules consumed.

The energy of light irradiance on the surface of the 4.0-PBR was calculated as follows:

Light intensity = 75 W/m^2 = 75 J/s/m^2

Time = 571 hours, corresponding to 2055600 s

Illuminated surface of the reactor = 355.34 cm^2, corresponding to 0.0355 m^2

Glass transparency = 0.80

Light dilution = 1.57

Energy of light irradiance = (75 J/s/m^2 × 2055600 s × 0.0355 m^2 × 0.80) / 1.57= 2788807.6 J

Energy of the consumed acetate = 12.66 (gr)/59 (MW) × 708.8 kJ/mol (heat of combustion of acetate) = 152091.7 J

Energy input = 2788807.6 J + 152091.7 J = 2940899.3 J

LCE = Energy output / Energy input = 21247.48 / 2940899.3 = 0.00722 ≈ 0.72%
